# Aneurysm Perforation Due to Advancement of the Coil Delivery Wire During Stent-Assisted Embolization

**DOI:** 10.7759/cureus.28063

**Published:** 2022-08-16

**Authors:** Takuya Osuki, Hiroyuki Ikeda, Minami Uezato, Masanori Kinosada, Masaki Chin

**Affiliations:** 1 Neurosurgery, Kurashiki Central Hospital, Kurashiki, JPN

**Keywords:** perforation, stent, lvis, delivery wire, coil, aneurysm

## Abstract

We report a case of intraprocedural aneurysm rupture during coil embolization caused by a coil delivery wire. A 68-year-old woman underwent stent-assisted coil embolization for an unruptured aneurysm in the internal carotid artery (ICA). A low profile visible intraluminal support device was deployed at the aneurysm neck. Coil embolization was performed with a jailing technique. After deflection of the jailed microcatheter was released, a coil was placed in the aneurysm against resistance to coil insertion. The movement of the microcatheter tip was restricted with the stent. A coil delivery wire that was advanced after coil detachment perforated the aneurysm. Hemostasis was achieved, and coil embolization was finished with a slight neck remnant. Complete occlusion of the aneurysm was confirmed on angiography six months later. Advancement of a coil alignment marker after coil detachment may cause aneurysm perforation due to coil delivery wire advancement. In stent-assisted coil embolization, when the movement of the microcatheter tip in the aneurysm is restricted by the stent and there is resistance to coil insertion, the risk of perforation due to the coil delivery wire after coil detachment should be noted.

## Introduction

Intraprocedural rupture is one of the most feared complications of endovascular surgery [1-3]. The recent incidence of intraprocedural rupture in unruptured aneurysms is reported to be as low as 0.9%-3.8% [4-11]. Perforations caused by coils, microcatheters, microguidewires, stents, and unknown origins are reported to be a mechanism of intraprocedural rupture [4-8]. However, perforations caused by coil delivery wires are uncommon. Herein, we report a case of intraprocedural rupture caused by coil delivery wire advancement.

## Case presentation

**Figure 1:**
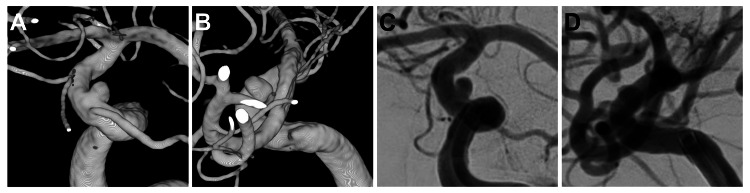
Working projection angiography before endovascular surgery. 3D reconstructed angiography (A, B) and angiography (C, D) of the left internal carotid artery. The angle was set so that the aneurysm neck and the parent vessel were separated (A, C), and the internal carotid artery in which the stent was to be placed was depicted long enough (B, D).

The patient was a 68-year-old woman with hypertension. She underwent neck clipping for subarachnoid hemorrhage due to right internal carotid artery (ICA) aneurysm rupture 23 years before and had no neurological deficit thereafter. Recurrence of the right ICA aneurysm and a de novo left ICA aneurysm were incidentally found by CT angiography. Cerebral angiography showed a recurrent aneurysm that was as large as 6.28 mm in the right ICA, and a de novo aneurysm that was as large as 2.72 mm in neck, 2.00 mm in height, and 3.47 mm × 3.68 mm in dome on the anterior lateral side of the left ICA C2 segment (Figure 1). The diameter of the left ICA was 3.70 mm × 3.68 mm at the proximal portion of the aneurysm (C3 portion), 3.64 mm × 3.52 mm at the aneurysm neck (C2 portion), and 2.92 mm × 2.81 mm at the distal portion of the aneurysm (C1 portion). The patient requested treatment of bilateral ICA aneurysms, and stent-assisted coil embolization for both aneurysms was scheduled. She took aspirin 100 mg/day and clopidogrel 75 mg/day for 14 days before treatment for the recurrent right ICA aneurysm. Two months after the treatment, the left ICA aneurysm was embolized. The VerifyNow system (Accumetrics, San Diego, CA, USA), a device that allows assessment of platelet reactivity to antiplatelet medications, showed aspirin resistance unit of 412 and P2Y12 reaction unit of 215, and cilostazol 200 mg/day was additionally administered.

**Figure 2:**
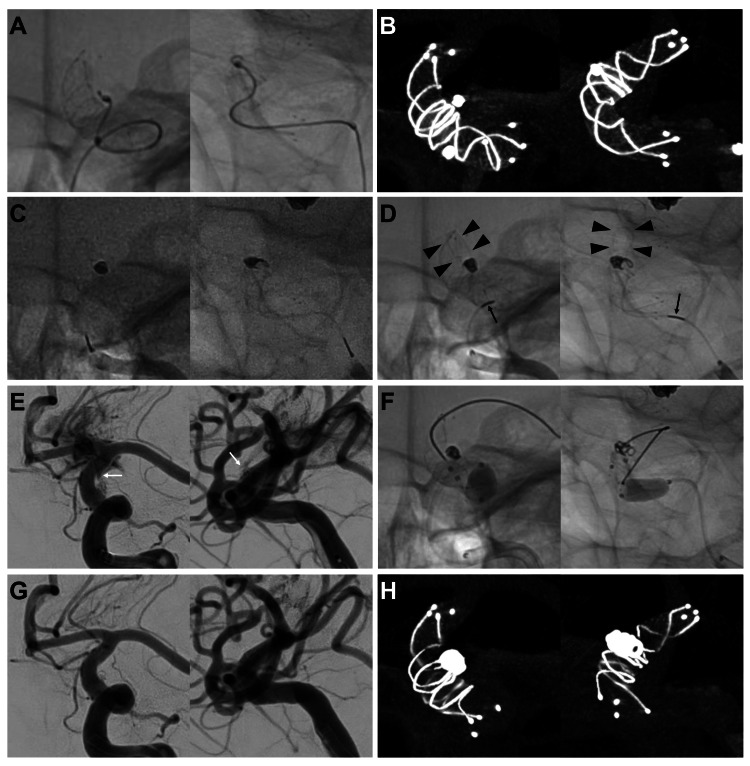
Imaging findings during endovascular surgery. The left side of each panel was the frontal working projection, and the right side of the lateral working projection. (A) Native imaging. A microcatheter was advanced into the aneurysm. One loop of a coil was inserted, and a stent was placed. (B) Cone beam CT with five-fold diluted contrast media shows an increased metal coverage of the aneurysm neck. (C) Native imaging. The coil was placed in the aneurysm, and the rear end of the coil alignment marker and the second microcatheter marker were matched. (D) Native imaging after coil detachment shows 9-mm advancement of the coil alignment marker (black arrows) compared to its position before detachment and advancement of the coil delivery wire (arrowheads) through the aneurysm wall. (E) Left internal carotid angiography after retrieving the perforated coil delivery wire showing extravasation of contrast media from the aneurysm (white arrow). (F) Native imaging. The balloon catheter was expanded at the proximal portion of the aneurysm. (G) Left internal carotid angiography after temporary balloon expansion shows no extravasation of contrast media from the aneurysm, and the aneurysm was occluded with a slight neck remnant. (H) Cone beam CT with five-fold diluted contrast media at the end of the surgery shows an increased metal coverage by shortening the proximal portion of the stent.

The patient received endovascular surgery under general anesthesia. We kept activated clotting time over 250 s during the surgery. An 8-Fr guiding catheter was inserted into the left femoral artery and was advanced to the cervical portion of the left ICA. Via this catheter, a 6-Fr Cerulean (Medikit, Tokyo, Japan) was advanced to the cavernous portion of the left ICA, and a microcatheter for stent deployment (Headway 21; Terumo, Tokyo, Japan) was placed at the superior trunk of the left middle cerebral artery. A microcatheter for coil embolization (Headway 17; Terumo, Tokyo, Japan), which was shaped with steam in accordance with the form of the aneurysm and the left ICA, was advanced into the aneurysm. After one loop of Target 360 Nano 2.5 mm × 4 cm detachable coil (Stryker Neurovascular; Fremont, CA, USA) was placed into the aneurysm through the microcatheter, a low-profile visible intraluminal support device (LVIS) Blue 3.5 mm × 22 mm (Terumo, Tokyo, Japan) was deployed from the C1 portion to the C4 portion of the left ICA with increased metal coverage at the aneurysm neck (Figure 2A). The Target 360 Nano 2.5 mm × 4 cm was removed, and cone beam CT with five-fold diluted contrast media showed good attachment of the stent to the vessel wall (Figure 2B). Insertion of the Target 360 Nano 2.5 mm × 4 cm into the aneurysm via the Headway 17 that was jailed with the stent was attempted, but it was retrieved because the resistance increased during the coil insertion. A smaller coil was chosen, and a Target 360 Nano 2.0 mm × 3 cm was placed in the aneurysm. The longer the coil was placed into the aneurysm, the greater the resistance became, but the coil was entirely placed in the aneurysm after deflection of the Headway 17 was gradually released (Figure 2C). Once the rear end of the coil alignment marker was overlapped to the second microcatheter marker, the coil delivery wire was fixed at the proximal part of the microcatheter, and the coil was detached. The coil alignment marker advanced 9 mm immediately after coil detachment, and a coil delivery wire perforated through the aneurysm wall (Figure 2D). Angiography after retrieving the coil delivery wire showed extravasation of contrast media from the aneurysm (Figure 2E). A Scepter XC balloon catheter 4 mm × 11 cm (Terumo, Tokyo, Japan) was advanced from the guiding catheter and expanded in the left ICA proximal to the aneurysm after antihypertensive therapy, manual common carotid artery compression, and reversal of heparin with protamine were implemented (Figure 2F). The Headway 17 came out from the aneurysm because the expansion of the Scepter balloon shortened the proximal side of the LVIS. Angiography 5 min after blocking the proximal ICA revealed the disappearance of extravasation of contrast media and a slight neck remnant of the aneurysm (Figure 2G). Coil insertion with a trans-cell approach was difficult because of an increased metal coverage of the aneurysm neck with the LVIS. Cone beam CT with five-fold diluted contrast media showed an increased metal coverage at the proximal portion of the aneurysm neck by the shortened LVIS. Angiography 10 min later revealed no extravasation of contrast media, and the surgery was finished.

**Figure 3:**
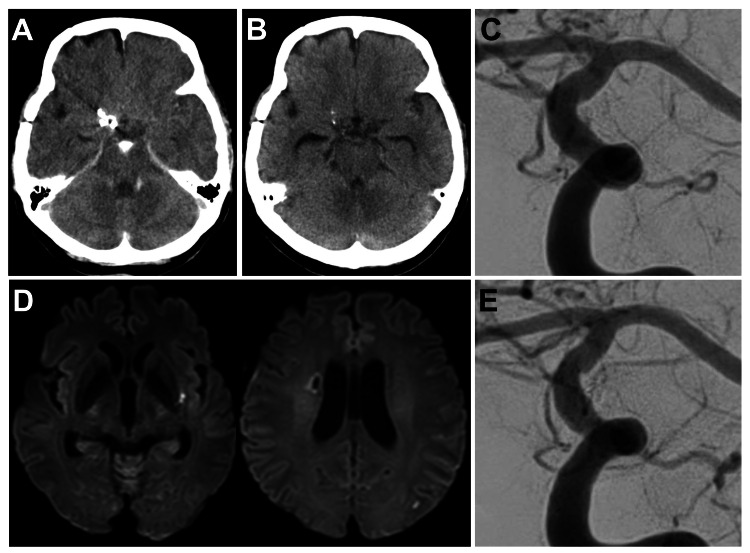
Imaging findings after endovascular surgery. (A) CT immediately after surgery shows a high-density area in the interpeduncular cistern. (B) CT the day after surgery revealed that the high-density area in the subarachnoid space has almost disappeared. (C) Left internal carotid angiography of working projection the day after surgery shows no extravasation of contrast media and a slight neck remnant of the aneurysm. (D) Diffusion-weighted MRI of the head the day after surgery shows a small high intensity spot outside the left putamen and in the left occipital lobe. (E) Left internal carotid angiography of working projection six months after surgery showing complete occlusion of the aneurysm.

The CT immediately after surgery showed a high-density area in the interpeduncular cistern (Figure 3A). The patient was kept under general anesthesia, and CT the day after surgery revealed the disappearance of most of the high-density area (Figure 3B). Angiography the day after surgery showed no extravasation of contrast media and a slight neck remnant of the aneurysm. There was no neurological deficit after emergence from general anesthesia. MRI revealed diffuse and spotted cerebral infarction in the left cerebral hemisphere (Figure 3D). The VerifyNow system showed an aspirin resistance unit of 418 and a P2Y12 reaction unit of 256, and oral administration of aspirin 100 mg/day and clopidogrel 75 mg/day were continued while cilostazol was discontinued because of the perforation. The postoperative course was uneventful, and the patient was discharged home 10 days after surgery. Angiography six months after surgery revealed complete occlusion of the aneurysm (Figure 3E).

## Discussion

**Figure 4:**
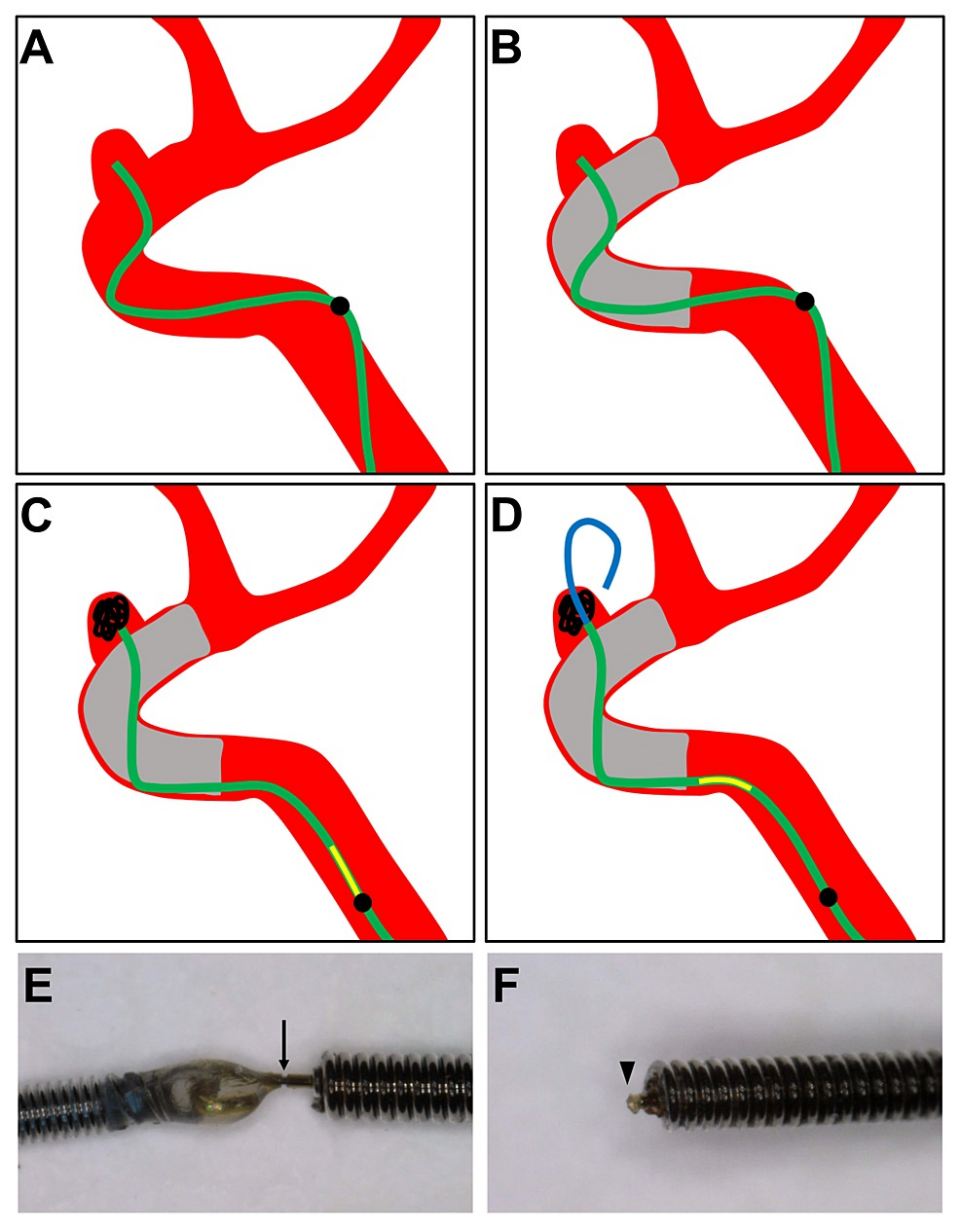
The process of aneurysm perforation. (A-D) The process of aneurysm perforation due to coil delivery wire advancement. Green line: the microcatheter for coil embolization, Black line: the coil, Yellow line: the coil alignment marker, Blue line: the perforated coil delivery wire, Black point: the second microcatheter marker, Grey area: the low-profile visible intraluminal support device (LVIS). (A) A microcatheter was inserted into the aneurysm, conforming to the shape of the blood vessel. (B) An LVIS was placed at the aneurysm neck. (C) The movement of the microcatheter tip was restricted by the LVIS. Deflection of the proximal portion of the microcatheter was released, and the microcatheter was straightened. A coil was inserted into the aneurysm against resistance. (D) After coil detachment, deflection of the coil delivery wire was released, and the aneurysm was perforated. (E-F) Photographs of the Target coil detachment zone. (E) The coil (0.010 inch) and the coil delivery wire (0.014 inch) are connected in the detachment zone (arrow). (F) The sharp tip (arrowhead) of the coil delivery wire after coil detachment.

In this case, first of all, a microcatheter for coil embolization was advanced into the aneurysm (Figure 4A). The microcatheter movement was restricted because it was jailed due to LVIS deployment (Figure 4B). Since there was resistance when placing a coil, the coil was inserted into the aneurysm while pulling the microcatheter (Figure 4C). The movement of the microcatheter tip was restricted because of an increased metal coverage of the aneurysm neck with the LVIS. On the other hand, the proximal portion of the microcatheter was flexible and straightened. There was resistance when placing the coil, and the coil delivery wire wound inside the microcatheter. During coil detachment, deflection of the coil delivery wire was released, and the wire was advanced and perforated the aneurysm (Figure 4D). Since the tip of the coil delivery wire became sharp after coil detachment, there was a high possibility of its causing perforation once being advanced toward the aneurysm wall (Figure 4E,F).

In this case, we used a jailing technique [12], which may have caused the advancement of the coil delivery wire during coil detachment because the microcatheter was jailed by the stent, and its movement was restricted. When using a semi-jailing technique [13] and a trans-cell approach [14], the microcatheter movement is not restricted by the stent, and the microcatheter moves backward when there is resistance to coil insertion. During coil detachment, the coil delivery wire does not advance, but the microcatheter does. Accordingly, among techniques for stent-assisted coil embolization, only the jailing technique is associated with perforation caused by coil delivery wire advancement.

In this case, an LVIS, a braided stent, was placed at the aneurysm neck with an increased metal coverage in anticipation of the flow diverter effect. An LVIS of 3.5 mm, which expands up to 3.7 mm, was selected based on the diameter of the parent vessel. The maximum diameter of the LVIS was larger than that of the parent vessel, and stent coverage was increased, which might have increased the radial force at the proximal portion of the aneurysm neck and restricted the microcatheter movement. On the other hand, since Neuroform Atlas (Stryker Neurovascular, Salt Lake Cty, UT, USA) and Enterprise (Codman Neurovascular, Miami Lakes, FL, USA), which are laser cut stents, are placed with an unsheath procedure, and the radial force of the stent is almost the same for the aneurysm neck and the proximal portion of the parent vessel. Thus, increasing the metal coverage of the braided stent at the aneurysm neck may restrict the movement of the microcatheter tip, and a coil delivery wire may easily advance during coil detachment. If the aneurysm is larger, the jailed microcatheter can be pulled in the aneurysm, the movement of the microcatheter tip is less restricted within the aneurysm, and the coil delivery wire can be advanced within the aneurysm, so this complication is unlikely to occur.

In this case, a smaller coil with different shapes should have been chosen because resistance to coil insertion increases due to the situation in which the movement of the microcatheter tip is restricted. Alternatively, removing the jailed microcatheter without the coil insertion in anticipation of the flow diverter effect may be an option. Using tactile and visual feedback to anticipate problems would have saved the complication in this case. During surgery, the advancement of a coil alignment marker was confirmed after coil detachment, but aneurysm perforation due to the coil delivery wire was not recognized. Angiography was performed to clarify the situation after the coil delivery wire was retrieved, and aneurysm perforation was recognized. When aneurysm perforation occurs, the device that caused the perforation should be left on site [15]. The coil delivery wire that perforated the aneurysm must be removed; however, additional coil insertion, blocking of the ICA, preparation of heparin reverse, and hypotensive management should have been performed before removal of the coil delivery wire. In this case, the high-density area in the subarachnoid space on postoperative CT was mostly due to leakage of contrast media, as it was washed away on CT the following day. Angiography immediately after removal of the coil delivery wire indicated that the contrast agent leaked from the perforation site, and if the situation had been recognized, angiography should not have been performed at the normal injection pressure.

## Conclusions

The advancement of a coil alignment marker after coil detachment may cause aneurysm perforation due to coil delivery wire advancement. In stent-assisted coil embolization, when the movement of the microcatheter tip in the aneurysm is restricted by the stent and there is resistance to coil insertion, the risk of perforation due to the coil delivery wire after coil detachment should be noted.
